# The complete chloroplast genome sequence of *Cyrtomium fortunei* (Dryopteridaceae), an important medical fern

**DOI:** 10.1080/23802359.2018.1443044

**Published:** 2018-02-27

**Authors:** Shufeng Li, Shanshan Liu, Zhen Wang, Ting Wang, Yingjuan Su

**Affiliations:** a School of Life Sciences, Sun Yat-sen University, Guangzhou, China;; b College of Life Sciences, Nanjing Agricultural University, Nanjing, China;; c College of Life Sciences, South China Agricultural University, Guangzhou, China;; d Research Institute of Sun Yat-sen University in Shenzhen, Shenzhen, China

**Keywords:** *Cyrtomium fortunei*, chloroplast genome, medical fern, phylogenetic analysis

## Abstract

The complete chloroplast genome of an important medical fern *Cyrtomium fortunei* has been sequenced. Its genome is 151,699 bp in length with a pair of inverted repeats (IRs, 23,875 bp), separated by a small single copy region (SSC, 21,625 bp) and a large single copy region (LSC, 82,324 bp). It contains 132 genes, covering 88 protein coding genes, 35 tRNA genes, eight rRNA genes, and one pseudogene. Maximum likelihood analysis indicates that the phylogenetic tree is monophyletic with three clades. *Cyrtomium fortunei* is closely related to *C. devexiscapulae*, which further forms a sister clade to *C. falcatum*.

*Cyrtomium fortunei* J. Smith (syn.: *Polystichum fortunei* Nakai) belongs to Dryopteridaceae and is known as holly fern. Its distinctive features are simply ovate-oblong pinnate leaves composed of dull green sickle-shaped pinnae with fairly smooth margins (Zhang et al. 2013). *Cyrtomium fortunei* is apomictic triploids (*n* = 2*n* = 123) (Takamiya 1996; Ootsuki et al. 2011), growing in limestone crevices in open areas or forests with altitude 100–2400 m (Zhang et al. 2013). The rhizomes of this fern, called Guan-zhong in China (China Food and Drug Administration 2015), have been widely used as a traditional medicinal plant for clearing heat and damp, cooling blood and hemostasis, and insecticide (Cui and Liu 2014). However, because there are great confusions in original plants of Guan-zhong, including other species in *Cyrtomium* and other ferns, mixes or misuses obviously occur (Cui and Liu 2014). Furthermore, the delineation of species within *Cyrtomium* and phylogenetic relationships of the genus in ferns are not well solved (Lu et al. 2005, 2007). Therefore, acquirement of the complete chloroplast of *C. fortunei* has double meaning for identification of medicinal plants and phylogenetic analysis.

The samples of *C. fortunei* were obtained from Medical Plants Garden in Guangdong Food and Drug Vocational College, China (23°20’85.47’’N, 113°38’0.99’’E). The specimen is stored in Herbarium of Sun Yat-sen University (SYS; voucher: *SS Liu 20161009*). We isolated DNA from leaf tissues, which was further broken into 300 bp fragment using Covaris M220 (Covaris Inc., MS, USA). After paired-end library was constructed, whole genome sequencing was performed on Illumina Hiseq 2500. Trimmomatic v0.32 and Fastqc v0.10.0 were used to filter and trim reads, and visualize the quality of the clean reads, respectively. We assembled the complete chloroplast genome using Velvet v1.2.07 (Zerbino and Birney 2008). Annotation was separately performed with DOGMA and tRNAscan-SE to predict protein-coding genes (PCGs), transfer RNA (tRNA) genes, and ribosome RNA (rRNA) genes (Lowe and Eddy 1997; Wyman et al. 2004). The phylogenetic analysis was conducted based on chloroplast genomes of 15 ferns. *Plagiogyria glauca* was used as outgroup. A maximum likelihood (ML) tree was constructed using RAxML v.8.0 (Stamatakis 2014) with 1000 bootstrap replicates.

The complete chloroplast genome of *C. fortunei* is 151,699 bp in length with a typical quadripartite structure containing a large single copy (LSC) region of 21,625 bp, a small single copy (SSC) region of 82,324 bp, and a pair of 23,875 bp inverted repeat (IRa and IRb) regions (GenBank accession number: MG913607). The genome encodes 132 genes, covering 88 PCGs, 35 tRNA genes, eight rRNA genes, and one pseudogene. Of them, 14 are duplicated, including four PCGs (*ycf2*, *psbA*, *rps7*, and *rps12*), six tRNA genes (*trnN*-*GUU*, *trnH*-*GUG*, *trnI*-*GAU*, *trnA*-*UGC*, *trnT-UGU*, and *trnR*-*ACG*), and four rRNA genes (*rrn5*, *rrn4.5*, *rrn23*, and *rrn16*). The chloroplast genome is the typical AT rich (57.6%). The ML phylogenetic tree is monophyletic with three independent clades. *Cyrtomium fortunei* is closely related to *C. devexiscapulae*, which further forms a sister clade to *C. falcatum* with a strong bootstrap value of 100% (Figure 1).

**Figure 1.**
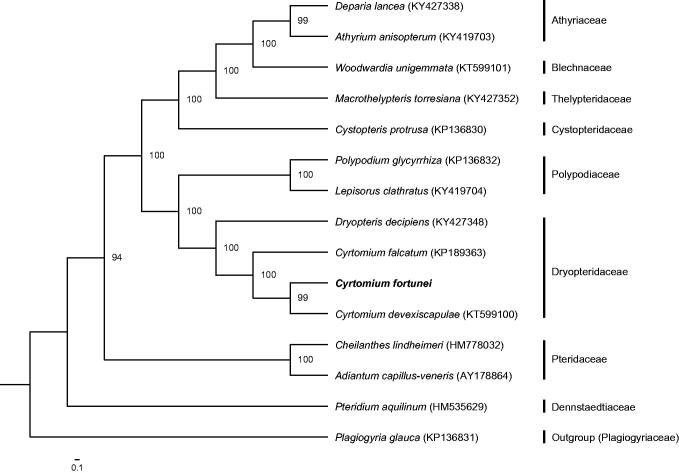
Phylogenetic tree based on whole chloroplast genomes of 15 ferns. These ferns can be divided into three independent clades with *Plagiogyria glauca* as outgroup. *Cyrtomium* is resolved as monophyletic. Maximum likelihood bootstrap support values for 1000 replicates are shown beside nodes.
